# Study on serum TL1A levels and their correlation with Th17 cells, IL-17 and IL-21 in children with Graves’ disease

**DOI:** 10.3389/fimmu.2024.1455025

**Published:** 2024-12-13

**Authors:** Lijun Hao, Jiong Yang, Biyao Lian, Chunyan Yin, Yanfeng Xiao, Yuesheng Liu

**Affiliations:** ^1^ Neonatal Department, Xi’an People’s Hospital (Xi’an Fourth Hospital), Xi’an, China; ^2^ Department of Pediatrics, The Second Affiliated Hospital of Xi’an Jiaotong University, Xi’an, Shaanxi, China

**Keywords:** Graves’ disease, children, TL1A, Th17 cells, IL-17, IL-21

## Abstract

**Objective:**

To investigate serum TL1A levels and their correlation with Th17 cells, IL-17, and IL-21 in children with Graves’ disease (GD).

**Methods:**

Thirty-seven children (12 males and 25 females) aged 9-14 years with newly diagnosed and untreated GD were enrolled in this study. Serum TL1A, IL-17, and IL-21 levels were measured using enzyme-linked immunosorbent assay (ELISA). The percentage of Th17 cells in peripheral blood was determined by flow cytometry. The correlation between serum TL1A levels and Th17 cells, IL-17, and IL-21 was analyzed using Pearson’s correlation coefficient.

**Results:**

Serum TL1A levels and the percentage of Th17 cells were significantly higher in children with GD compared to healthy controls (P<0.05). Serum IL-17 and IL-21 levels were also significantly elevated in GD patients (P<0.05). Serum TL1A levels positively correlated with the percentage of Th17 cells (r=0.625, P<0.05), IL-17 (r=0.573, P<0.05), and IL-21 (r=0.542, P<0.05) in children with GD.

**Conclusion:**

Serum TL1A levels are increased in children with GD and positively correlate with Th17 cells, IL-17, and IL-21, suggesting that TL1A may play a role in the pathogenesis of GD by regulating Th17 cell differentiation and the production of IL-17 and IL-21.

## Introduction

Graves’ disease (GD) is an autoimmune thyroid disorder characterized by hyperthyroidism, goiter, and ophthalmopathy. It is the most common cause of hyperthyroidism in children and adolescents, with a peak incidence between 11 and 15 years of age (1). The pathogenesis of GD involves complex interactions between genetic, environmental, and immunological factors, leading to the production of autoantibodies against the thyroid-stimulating hormone receptor (TSHR) (2). These autoantibodies, known as TSHR autoantibodies (TRAb), bind to and stimulate the TSHR, causing excessive thyroid hormone production and the clinical manifestations of hyperthyroidism (3).

Recent studies have highlighted the role of T helper 17 (Th17) cells in the pathogenesis of autoimmune diseases, including GD (4). Th17 cells are a subset of CD4+ T cells that produce interleukin-17 (IL-17), a pro-inflammatory cytokine involved in the regulation of immune responses and tissue inflammation (5). IL-17 has been shown to promote the production of other pro-inflammatory cytokines, chemokines, and matrix metalloproteinases, contributing to the recruitment and activation of immune cells and the destruction of target tissues (6). In addition to IL-17, Th17 cells also produce interleukin-21 (IL-21), a cytokine that promotes Th17 cell differentiation and enhances IL-17 production, creating a positive feedback loop (7).

Tumor necrosis factor-like cytokine 1A (TL1A), also known as TNF superfamily member 15 (TNFSF15), is a recently identified member of the TNF superfamily (8). TL1A is expressed by various cell types, including macrophages, dendritic cells, and T cells, and has been implicated in the pathogenesis of several autoimmune diseases, such as inflammatory bowel disease, rheumatoid arthritis, and psoriasis (9–11). TL1A exerts its effects by binding to its receptor, death receptor 3 (DR3), which is expressed on activated T cells, particularly Th17 cells (12). The interaction between TL1A and DR3 has been shown to promote Th17 cell differentiation, enhance IL-17 production, and exacerbate autoimmune inflammation (13).

Despite the growing evidence supporting the role of TL1A and Th17 cells in autoimmune diseases, their involvement in the pathogenesis of GD, particularly in children, remains largely unknown. Therefore, this study aimed to investigate serum TL1A levels and their correlation with Th17 cells, IL-17, and IL-21 in children with newly diagnosed and untreated GD, to better understand the potential role of TL1A in the pathogenesis of GD.

## Materials and methods

### Study population

Thirty-seven children (12 males and 25 females) aged 9-14 years with newly diagnosed and untreated GD were enrolled in this study. The diagnosis of GD was based on clinical manifestations (hyperthyroidism, goiter, and/or ophthalmopathy), laboratory findings (elevated thyroid hormone [FT4, TT4, FT3, TT3] and suppressed thyroid-stimulating hormone [TSH] levels), and the presence of TRAb. Age- and sex-matched healthy children with no personal or family history of autoimmune diseases were included as controls. Exclusion criteria for both groups were the presence of other autoimmune diseases(including but not limited to inflammatory bowel disease, rheumatoid arthritis, and type 1 diabetes), acute or chronic infections, malignancies, and the use of immunosuppressive or immunomodulatory drugs. The study was approved by the ethics committee of the Second Affiliated Hospital of Xi’an Jiaotong University (No.2024094). The study was conducted in accordance with the Declaration of Helsinki. All parents or guardians signed informed consent forms before the enrollment of the children. This was a prospective, cross-sectional study conducted between February and October 2024. Patients were recruited and samples were collected after obtaining ethics approval. The parameters measured in this study (TL1A, IL-17, IL-21, and Th17 cell percentages) are not routinely measured in clinical practice but were specifically analyzed for research purposes.

### Sample collection and processing

Venous blood samples were collected from all participants after an overnight fast. Serum was separated by centrifugation at 1500 × g for 10 minutes at room temperature and stored at -80°C until analysis. Peripheral blood mononuclear cells (PBMCs) were isolated from heparinized blood samples using Ficoll-Hypaque density gradient centrifugation (Lymphoprep, Axis-Shield, Oslo, Norway) for flow cytometric analysis.

### Measurement of serum TL1A, IL-17, and IL-21 levels

Serum TL1A, IL-17, and IL-21 levels were measured using commercially available enzyme-linked immunosorbent assay (ELISA) kits (R&D Systems, Minneapolis, MN, USA) according to the manufacturer’s instructions. Briefly, serum samples were diluted and added to the wells of microtiter plates coated with capture antibodies specific for TL1A, IL-17, or IL-21. After incubation and washing, biotinylated detection antibodies were added, followed by streptavidin-horseradish peroxidase (HRP) conjugate. The plates were then developed with tetramethylbenzidine (TMB) substrate, and the optical density was measured at 450 nm using a microplate reader (Bio-Rad, Hercules, CA, USA). The concentrations of TL1A, IL-17, and IL-21 were calculated using standard curves generated with recombinant human proteins. The sensitivity of the assays was 5.0 pg/mL for TL1A, 0.5 pg/mL for IL-17, and 4.8 pg/mL for IL-21. Intra- and inter-assay coefficients of variation were <10% for all assays.

### Flow cytometric analysis of Th17 cells

The percentage of Th17 cells in peripheral blood was determined by flow cytometry. PBMCs were stimulated with phorbol 12-myristate 13-acetate (PMA; 50 ng/mL) and ionomycin (1 μg/mL) in the presence of brefeldin A (10 μg/mL) for 4 hours at 37°C in a humidified atmosphere containing 5% CO2. After stimulation, cells were stained with fluorochrome-conjugated antibodies against surface markers CD4 and CD45RO (BD Biosciences, San Jose, CA, USA) for 30 minutes at 4°C in the dark. Following surface staining, cells were fixed and permeabilized using the Cytofix/Cytoperm kit (BD Biosciences) according to the manufacturer’s instructions. Intracellular staining was then performed using fluorochrome-conjugated antibodies against IL-17A (BD Biosciences). Isotype-matched control antibodies were used to determine background staining. Stained cells were acquired on a FACSCalibur flow cytometer (BD Biosciences) and analyzed using FlowJo software (TreeStar, Ashland, OR, USA). The percentage of CD4+CD45RO+IL-17A+ cells within the CD4+ T cell population was considered as the Th17 cell percentage.

### Statistical analysis

Data were expressed as mean ± standard deviation (SD), median (interquartile range), or percentages as appropriate. The normality of data distribution was assessed using the Kolmogorov-Smirnov test. Comparisons between groups were performed using the Student’s t-test or Mann-Whitney U test for continuous variables and the chi-square test or Fisher’s exact test for categorical variables. Correlations between serum TL1A levels and Th17 cells, IL-17, and IL-21 were analyzed using Pearson’s correlation coefficient or Spearman’s rank correlation coefficient, depending on the normality of data distribution. The correlations between TL1A, IL-17, IL-21 and clinical parameters (T3, T4, TSH, TRAb) were analyzed within the GD group using Spearman’s correlation coefficient. A P-value <0.05 was considered statistically significant. Statistical analyses were performed using SPSS version 25.0 (IBM Corp., Armonk, NY, USA) and GraphPad Prism version 8.0 (GraphPad Software, La Jolla, CA, USA).

## Results

### Clinical characteristics of the study population

The clinical characteristics of the study population are summarized in **Table 1**. There were no significant differences in age and sex between the GD and control groups. As expected, children with GD had significantly higher FT4, TT4, FT3, TT3 levels and lower TSH levels compared to healthy controls (P<0.05). The median TRAb levels was also significantly higher in the GD group (P<0.05). The prevalence of goiter and ophthalmopathy was 83.3% and 27.8%, respectively, in children with GD.

**Table 1:** Clinical characteristics of the study population.

Parameter	GD group (n=37)	Control group (n=37)	P-value
Age (years)	11.5 ± 1.7	11.8 ± 1.5	0.442
Sex (male/female)	12/25	12/25	1.000
FT4 (pmol/L)	45.6 ± 12.3	14.2 ± 2.1	<0.05
TT4 (pmol/L)	238 ± 29.7	95 ± 12.4	<0.05
FT3 (pmol/L)	26.5 ± 4.4	6.1 ± 1.2	<0.05
TT3 (pmol/L)	6.1 ± 1.6	2.4 ± 0.6	<0.05
TSH (mIU/L)	0.01 (0.01-0.03)	2.36 (1.54-3.27)	<0.05
TRAb (IU/L)	12.5 (8.2-18.9)	0.4 (0.2-0.6)	<0.05
Goiter, n (%)	30 (81.1)	0 (0)	<0.05
Ophthalmopathy, n (%)	10 (27.0)	0 (0)	0.001

Data are expressed as mean ± SD, median (interquartile range), or number (percentage).

FT4, free thyroxine; TT4, total thyroxine; FT3, free triiodothyronine; TT3, total triiodothyronine; TSH, thyroid-stimulating hormone; TRAb, TSH receptor autoantibodies.

### Serum TL1A levels in children with GD

Serum TL1A levels were significantly higher in children with GD compared to healthy controls (**Table 2**). The median serum TL1A level in the GD group was 385.6 pg/mL (interquartile range: 278.4-512.3 pg/mL), while in the control group, it was 165.3 pg/mL (interquartile range: 118.9-220.7 pg/mL) (P<0.05). Within the GD group, we found no significant difference in serum TL1A levels between patients with and without ophthalmopathy (p = 0.32). However, the small number of patients with ophthalmopathy (n=10) limits the power of this analysis.

**Table 2:** Serum TL1A, IL-17, and IL-21 levels and Th17 cell percentage in the study population.

Parameter	GD group (n=37)	Control group (n=37)	P-value
TL1A (pg/mL)	385.6 (278.4-512.3)	165.3 (118.9-220.7)	<0.05
Th17 cells (%)	2.9 (2.1-3.6)	1.2 (0.8-1.5)	<0.05
IL-17 (pg/mL)	18.4 (13.2-25.6)	7.2 (4.9-10.1)	<0.05
IL-21 (pg/mL)	42.7 (31.5-57.3)	18.9 (13.6-25.2)	<0.05

Data are expressed as median (interquartile range). Statistical significance was determined using Mann-Whitney U test.

TL1A, tumor necrosis factor-like cytokine 1A; IL-17, interleukin-17; IL-21, interleukin-21.

### Th17 cell percentage in children with GD

**Figure 1:**
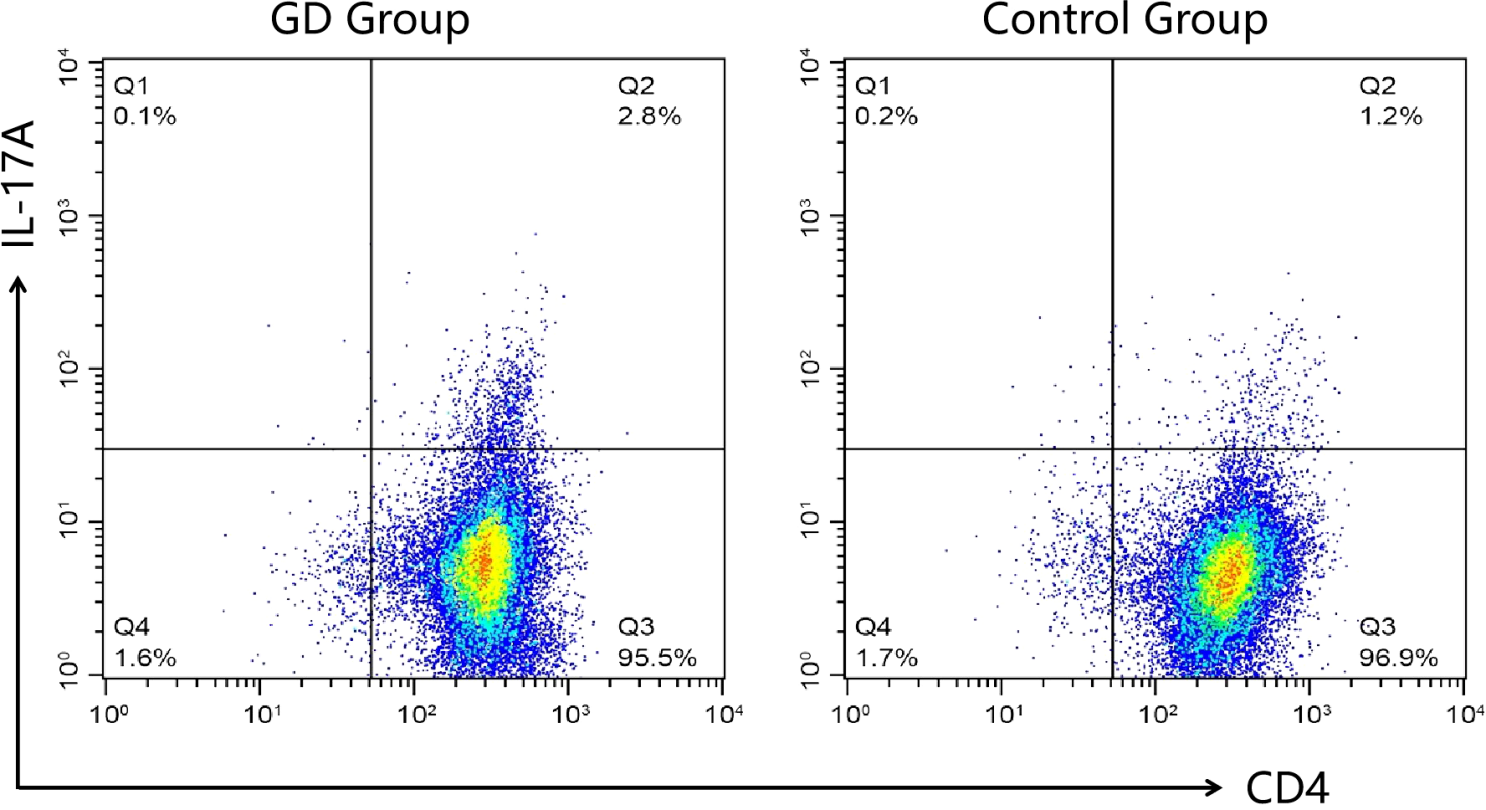
Representative flow cytometry plots of Th17 cell proportions in each group. Peripheral blood mononuclear cells from GD patients (GD group) and healthy controls (Control Group) were analyzed for CD4 and IL-17A expression. The numbers in the upper right quadrants indicate the percentage of CD4+IL-17A+ cells, representing the Th17 cell population. The GD group demonstrated a higher percentage of Th17 cells compared to the Control Group.

The percentage of Th17 cells in peripheral blood was significantly higher in children with GD compared to healthy controls (**Table 2**, **Figure 1**). The median Th17 cell percentage in the GD group was 2.9% (interquartile range: 2.1-3.6%), while in the control group, it was 1.2% (interquartile range: 0.8-1.5%) (P<0.05).

### Serum IL-17 and IL-21 levels in children with GD

Serum IL-17 and IL-21 levels were significantly elevated in children with GD compared to healthy controls (**Table 2**). The median serum IL-17 level in the GD group was 18.4 pg/mL (interquartile range: 13.2-25.6 pg/mL), while in the control group, it was 7.2 pg/mL (interquartile range: 4.9-10.1 pg/mL) (P<0.05). Similarly, the median serum IL-21 level in the GD group was 42.7 pg/mL (interquartile range: 31.5-57.3 pg/mL), while in the control group, it was 18.9 pg/mL (interquartile range: 13.6-25.2 pg/mL) (P<0.05).

### Correlation between serum TL1A levels and Th17 cells, IL-17, and IL-21 in children with GD

Serum TL1A levels positively correlated with the percentage of Th17 cells, serum IL-17 levels, and serum IL-21 levels in children with GD (**Table 3**). The correlation coefficients were 0.625 for Th17 cells (P<0.05), 0.573 for IL-17 (P<0.05), and 0.542 for IL-21 (P<0.05).

**Table 3:** Correlation between serum TL1A levels and Th17 cells, IL-17, and IL-21 in children with GD.

Parameter	Correlation coefficient (r)	P-value
Th17 cells (%)	0.625	<0.05
IL-17 (pg/mL)	0.573	<0.05
IL-21 (pg/mL)	0.542	<0.05

TL1A, tumor necrosis factor-like cytokine 1A; IL-17, interleukin-17; IL-21, interleukin-21.

Within the GD group, correlation analysis revealed significant positive correlations between TRAb levels and serum levels of TL1A (r=0.512, P<0.05) and IL-17 (r=0.483, P<0.05). However, no significant correlations were found between these cytokines and thyroid function parameters (FT3, FT4, and TSH) (all P>0.05) (**Table 4**).

**Table 4:** Correlation between serum TL1A, IL-17, IL-21 levels and clinical parameters in children with GD.

Parameter	TL1A		IL-17		IL-21	
	r	P-value	r	P-value	r	P-value
FT3	0.184	0.283	0.165	0.137	0.142	0.408
FT4	0.204	0.131	0.193	0.259	0.177	0.102
TSH	-0.167	0.099	-0.159	0.357	-0.116	0.096
TRAb	0.512	0.024	0.483	0.031	0.265	0.112

Correlation coefficients (r) were calculated using Spearman’s correlation analysis.

FT3, free triiodothyronine; FT4, free thyroxine; TSH, thyroid-stimulating hormone; TRAb, TSH receptor autoantibodies.

## Discussion

This study demonstrates that serum TL1A levels are significantly increased in children with newly diagnosed and untreated GD compared to healthy controls. Furthermore, serum TL1A levels positively correlate with the percentage of Th17 cells, IL-17, and IL-21 in children with GD. These findings suggest that TL1A may play a role in the pathogenesis of GD by regulating Th17 cell differentiation and the production of IL-17 and IL-21.

Th17 cells have been implicated in the pathogenesis of various autoimmune diseases, including GD (4). In the present study, we found that the percentage of Th17 cells was significantly higher in children with GD compared to healthy controls, consistent with previous reports (14, 15). Th17 cells produce IL-17 and IL-21 (5, 6).Previous studies showed that elevated levels of serum IL-17 and IL-21 were closely related to the onset of GD and Graves’ ophthalmopathy (GO), and were positively correlated with the clinical activity of GO (16). Zheng L et al. (17) found that IL-17 mRNA expression and IL-17 protein levels were significantly elevated in PBMCs of GD adults,which regulated by IL-23/IL-17 axis, not dependent on thyroid function. In this study, we found that serum levels of IL-17 and IL-21 were significantly elevated in children with GD compared to healthy controls, further supporting the involvement of Th17 cells in the pathogenesis of GD.

TL1A, a member of the TNF superfamily, has been shown to promote Th17 cell differentiation and enhance IL-17 production in various autoimmune diseases (9–11). The interaction between TL1A and its receptor, DR3, has been identified as a key pathway in the regulation of Th17 responses (12, 13). In the present study, we found that serum TL1A levels were significantly elevated in children with GD and positively correlated with the percentage of Th17 cells, IL-17, and IL-21. These findings suggest that TL1A may contribute to the pathogenesis of GD by promoting Th17 cell differentiation and the production of IL-17 and IL-21.

The exact mechanisms by which TL1A promotes Th17 cell differentiation and cytokine production in GD remain to be elucidated. However, previous studies have shown that TL1A enhances the expression of the transcription factor RORγt, which is essential for Th17 cell differentiation (18). Additionally, TL1A has been shown to activate the NF-κB and MAPK signaling pathways, which are involved in the regulation of pro-inflammatory cytokine production (19). Furthermore, TL1A may also contribute to the pathogenesis of GD by promoting the survival and proliferation of activated T cells, as well as enhancing the cytotoxic activity of CD8+ T cells (20, 21).

The findings of this study have potential clinical implications. Serum TL1A levels may have potential as a biomarker in GD. Future studies incorporating measures of disease severity are needed to evaluate its utility in assessing disease activity and predicting outcomes in children with GD. Moreover, targeting the TL1A-DR3 pathway may represent a promising therapeutic strategy for the treatment of GD and other Th17-mediated autoimmune diseases. Several approaches, such as neutralizing antibodies against TL1A or DR3, small molecule inhibitors of the TL1A-DR3 interaction, and downstream signaling inhibitors, are currently under investigation (22, 23). Our findings suggest several potential pathways through which TL1A may contribute to GD pathophysiology. TL1A binding to DR3 on T cells could activate NF-κB and MAPK signaling pathways, promoting Th17 cell differentiation and cytokine production. Additionally, TL1A may enhance RORγt expression, a key transcription factor for Th17 cells. Further mechanistic studies are needed to confirm these hypotheses.

Ren X et al. (24) found that TRAb levels were significantly positively correlated with the percentages of Th17 cells and IL-17 concentrations. However, there was no correlation between Th17/IL-17levels and FT3, FT4, TSH, TPOAb, and TGAb. Similarly, our study revealed significant positive correlations between TRAb levels and serum levels of TL1A, and IL-17 in children with GD, while no correlations were found with thyroid function parameters (FT3, FT4, and TSH). KeiichiTorimoto et al. (15) found that the percentage of activated Th17 cells positively correlated with not only TRAb but also FT3 and FT4. We speculate that the different results of the correlation between FT3, FT4 and TSH may have the following reasons: the immune environment of children is different from that of adults, and the different duration of onset may lead to varying degrees of imbalance in the immune homeostasis of the body. The upstream immune mechanism that regulates the number and function of Th17 cells is currently complex. previous studies found that the imbalance of intestinal microbiota, IL23/IL17 axis, and IL21 all affected Th17 cells and their cytokines (17, 25, 26). The strong correlation between these cytokines and TRAb, but not thyroid hormones, suggests that TL1A and Th17-related cytokines may be more closely associated with the autoimmune process rather than the severity of thyroid dysfunction in pediatric GD.

This study has several strengths, including the well-characterized cohort of children with newly diagnosed and untreated GD, the age- and sex-matched healthy control group, and the comprehensive assessment of Th17-related cytokines and cells. However, there are also some limitations that should be acknowledged. First, the sample size was relatively small, and larger studies are needed to confirm our findings. Second, the cross-sectional design of the study does not allow for the determination of causality between TL1A and Th17 responses in GD. Longitudinal studies are required to evaluate the temporal relationship between TL1A levels and disease activity. Third, we did not assess the expression of DR3 on Th17 cells or other immune cell subsets, which may provide further insights into the mechanisms of TL1A-mediated Th17 responses in GD.

In conclusion, this study demonstrates a significant association between elevated serum TL1A levels and increased Th17 cells, IL-17, and IL-21 in children with newly diagnosed and untreated GD. While these findings suggest a potential role for TL1A in GD pathogenesis, possibly through its association with Th17 cell differentiation and cytokine production, further research is needed to establish causal relationships and elucidate the precise mechanisms involved.

## Data Availability

The raw data supporting the conclusions of this article will be made available by the authors, without undue reservation.
